# Correlation between fecal calprotectin levels, disease severity and the hypervirulent ribotype 027 strain in patients with *Clostridium difficile* infection

**DOI:** 10.1186/s12879-016-1618-8

**Published:** 2016-06-22

**Authors:** Avi Peretz, Linda Tkhawkho, Nina Pastukh, Diana Brodsky, Chen Namimi Halevi, Orna Nitzan

**Affiliations:** Clinical Microbiology Laboratory, Baruch Padeh Medical Center, Poria, Tiberias, Israel; Faculty of Medicine, Bar Ilan University, Galilee, Israel; The Ruth and Bruce Rappaport Faculty of Medicine, Haifa, Israel; Unit of Infectious Diseases, Baruch Padeh Medical Center, Poria, Tiberias, Israel

**Keywords:** Calprotectin, *Clostridium difficile*, Ribotype 027, Clostridium severity score index

## Abstract

**Background:**

*Clostridium difficile* is the most common infectious etiology of nosocomial diarrhea. Fecal calprotectin (fc) is a sensitive marker of intestinal inflammation, found to be associated with enteric bacterial infections and inflammatory bowel disease.

**Methods:**

We evaluated fc levels using a Chemiluminescent immunoassay method, in hospitalized patients with *C. difficile* infection (CDI) diagnosed by molecular stool examination and assessed correlation with virulent ribotype 027 strain infection, antibiotic susceptibility by gradient Etest strip performed on *C. difficile* colonies and clinical and laboratory measures of disease severity. Statistical analysis was performed for correlation of fc levels with clinical and laboratory parameters, disease severity and patient outcomes.

**Results:**

Overall 29 patients with CDI were admitted at the Poria medical center in northern Israel, during June 2014-May 2015. Resistance to metronidazole was found in 3 (10.3 %) isolates and to vancomycin in 5 (17.2 %) isolates. Regarding patient outcomes, within 30 days of CDI diagnosis, recurrence of disease occurred in 10 (34.5 %) patients and 2 patients (6.9 %) died. Seven (24.1 %) isolates were *C. difficile* ribotype 027. Mean fc level was 331.4 μg/g (21–932). Higher fc levels were found in patients with *C. difficile* ribotype 027 (*p* < 0.0005). Fc levels were also correlated with elevated peripheral blood white cell count (*p* = 0.0007). A trend for higher fc levels was found in patients with a higher clostridium severity score index (*p* = 0.0633). No correlation was found between fecal calprotectin levels and age, sex, functional status, community versus hospital acquired CDI, antibiotic susceptibility, fever, and creatinine levels.

**Conclusions:**

Our study highlights the fact that fc has a potential role as a biomarker of disease severity and binary toxin producing ribotype associated disease.

## Background

*Clostridium difficile* (*C. difficile*) is the most common infectious etiology of nosocomial diarrhea in acute care settings, accounting for 15–25 % of all cases of antibiotic-induced diarrhea. *C. difficile* infection (CDI) is caused by toxigenic *C. difficile* that usually produces 2 major toxins – toxin A, an enterotoxin, and toxin B, a potent cytotoxin [1–3]. The emergence of a hyper-virulent clone of *C. difficile,* ribotype 027, in North America and Europe in the 2000s has caused numerous outbreaks in the healthcare setting [4]. Most patients develop diarrhea during or shortly after starting antibiotics. However, 25–40 % of patients may not become symptomatic for as long as 10 weeks after completing antibiotic therapy. Symptoms range from mild watery diarrhea and cramping abdominal pain, to fulminant colonic infection with complications of toxic megacolon, perforation and death. Several scoring systems that predict disease prognosis and aid in treatment decisions have been developed, including the clostridium severity score index that incorporates 9 clinical and laboratory variables. Antibiotic treatment of CDI consists of metronidazole in mild cases and vancomycin in moderate to severe infection, although treatment failures and recurrence of disease occur often [5, 6]. Fecal calprotectin (fc) is a sensitive marker of intestinal inflammation, and was found to be associated with enteric bacterial infections and inflammatory bowel disease [7]. Calprotectin is a 24 kDa dimer of calcium binding proteins S100A8 and S100A9. The complex accounts for up to 60 % of the soluble protein content of the neutrophil cytosol. In vitro studies show that calprotectin has bacteriostatic and fungistatic properties that arise from its ability to sequester manganese and zinc [8, 9].

The aim of our study was to evaluate fc levels in hospitalized patients with CDI and to assess if fc levels are correlated with ribotype 027 strain infection, with reduced susceptibility of the infectious strain to metronidazole or vancomycin, with clinical and laboratory tools of disease severity, as calculated by the Clostridium severity score index.

## Results

Out of 29 patients with CDI, 15 were females and 14 were males. Mean age was 64.5 years (17–95). Fifteen patients (51.7 %) were functionally debilitated and 5 (17.2 %) were nursing home residents. Thirteen patients (44.8 %) were community acquired cases and 16 patients (55.2 %) represented nosocomial CDI. In 24 cases the Clostridium severity score index was ≤3, and a score of 4–6 points was calculated in 5 patients. Reduced susceptibility to metronidazole was found in 3 (10.3 %) isolates and to vancomycin in 5 (17.2 %) isolates. Regarding patient outcomes, within 30 days of CDI diagnosis, recurrence of disease occurred in 10 (34.5 %) patients, and 2 patients (6.9 %) had died. One patient (3.4 %) developed a toxic megacolon. Seven (24.1 %) isolates were identified as *C. difficile* ribotype 027. Among the 13 community acquired patients with CDI, only 2 had the 027 ribotype, and among the 16 nosocomial cases, 5 were 027 ribotype positive. The mean severity score for ribotype 027 patients was 2.86 versus 1.95 in ribotype 027 negative patients.

**Fig. 1:**
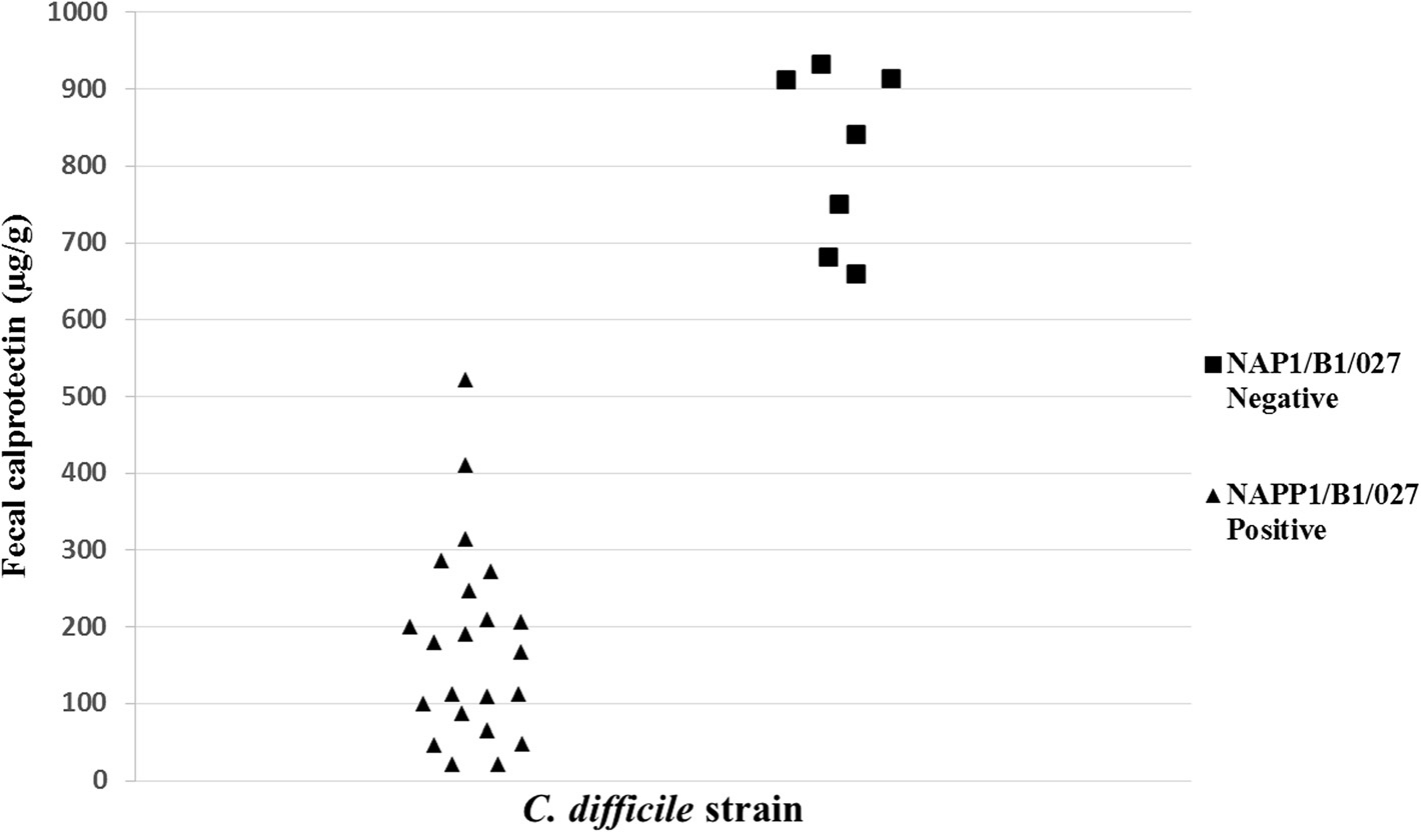
Fecal Calprotectin levels in patients with *Clostridium difficile* infection. and correlation with fecal Calprotectin levels

Mean calprotectin level was 331.4 μg/g (21–932). We performed assessment of the correlation between fecal calprotectin levels and clinical and laboratory parameters. We found higher calprotectin levels in patients with *C. difficile* ribotype 027, with a mean level of 811.9 μg/g (659–932), compared to patients with other *C. difficile* ribotypes, with a mean level of 178.6 μg/g (21–522), *p* < 0.0005 (Fig. 1). A significantly positive correlation of calprotectin levels was found with elevated peripheral blood white cell count (*p* = 0.0007). A trend for higher calprotectin levels was found in patients with a higher clostridium severity score index (*p* = 0.0633). No correlation was found between fecal calprotectin levels and age, sex, functional status, community versus nosocomial acquired CDI, antibiotic susceptibility, fever, and creatinine levels (Table 1). No correlation was found between fc levels and disease recurrence or mortality at 30 days, with a mean level of 284.7 μg/g (46–840) versus 356.1 μg/g (21–932) in those with and without recurrence, and 579.5 μg/g (248–911) versus 313.1 μg/g (21–932) in the patients who died versus those that survived (*p* = 0. 6662 and *p* = 0.255, respectively.).

**Table 1:** Clinical and laboratory parameters of patients with *Clostridium difficile* infection and correlation with fecal Calprotectin levels

Parameter	Mean (percent/range)	fc levels (μg/g), mean (range)	*P* -value for correlation with fc levels
Antibiotic-reduced susceptibility			
Metronidazole	3 (10.3 %)	Reduced susceptibility to Metronidazole: 177.7 (46–287)	0.5474
		Metronidazole susceptible 349 (21–932)	
Vancomycin	5 (17.2 %)	Reduced susceptibility to Vancomycin 336.8 (113–932)	0.7316
		Vancomycin susceptible 330.3 (21–912)	
Gender			
Male	15 (51.7 %)	340.8 (48–932)	
Female	14 (48.3 %)	321.4 (21–911)	0.7622
Age (years)	64.5 (17–95)		0.2764
Functional status			
Independent	14 (48.3 %)	316.8 (21–932)	
Debilitated	15 (51.7 %)	45.1 (46–911)	0.6815
CDI acquisition			
Community	10 (34.5 %)	273.6 (21–911)	
Nursing home	3 (10.3 %)	234.7 (21–411)	
Hospital	16 (55.2 %)	385.8 (46–932)	0.5678
Body temperature (°C)	37.0 (36–39.4)		0.8772
Peripheral white blood cell count (/μl)	13,363 (3070–43,210)		0.0007
Creatinine(g/dL)	1.3 (0.2–6.7)		0.2
Disease recurrence in 30 days	10 (34.5 %)	Disease recurrence 284.7 (46–800)	0.6662
		No recurrence 356.1 (21–932)	
30 day mortality			
Yes	2 (6.9 %)	579.5 (248–911)	
No	27 (93.1 %)	313.3 (21–932)	0.2550
*Clostridium* severity score index	2.17 (1–6)		0.0633

## Discussion

*C. difficile* is the leading infectious cause of antibiotic-associated diarrhea. CDI symptoms range from mild watery diarrhea to severe colitis, toxic megacolon, colonic perforation, and death. Guidelines for treatment of CDI stratify antibiotic treatment according to disease severity [6]. Currently there are several scoring systems, these consist of non-specific clinical and laboratory parameters that, as a whole, estimate disease severity and patient prognosis [10–12]. The search for specific markers indicative of disease severity is of great importance. As an example, in one study it was found that elevated PCT levels are associated with the severe CDI [13].

Recently, a few studies found that fecal calprotectin levels are associated with acute bacterial gastroenteritis [14, 15], despite the fact that up to now fc was used only to stratify between inflammatory and non-inflammatory bowel diseases. Calprotectin is a 24 kDa dimer of calcium binding proteins that accounts for up to 60 % of the soluble protein content of the neutrophil cytosol. Several recent studies found that fc levels are elevated in patients with CDI, compared with patients with non-*C. difficile* antibiotic-associated diarrhea and with patients with *C. difficile* colonization only [16,17]. In this study we aimed to find if fc levels are correlated with CDI severity and with disease caused by ribotype 027; this specific strain is associated with higher morbidity and mortality rates and increased antibiotic resistance. We found that fc levels were significantly associated with *C. difficile* ribotype 027. In routine practice most microbiology laboratories do not regularly assess *C. difficile* ribotype because the diagnosis is based on ELISA toxin assays, which don’t identify ribotypes. However, the elevated fc levels might alert clinicians to the possibility of a more severe and complicated course of disease that is associated with this ribotype. This may also affect empiric choice of the antibiotic therapy. For example, fidaxomicin treatment decreases recurrence rates in CDI, but not in cases of ribotype 027 associated disease [18]. Another study of 164 patients with CDI found a non-significant correlation of fc levels and ribotype 027 [16]. This might be due to analysis of fc levels in 4-tier percentile categories and not as a linear value as done in our study. We also found fc levels to be significantly associated with a higher peripheral blood white cell count, which might reflect an elevated degree of intestinal inflammation that is associated with fc stool levels. In this study there was a trend towards higher fc levels in patients with more severe disease as assessed by the clostridium severity score index. Statistical significance may not have been achieved due to the relatively small sample size. One other study assessed the correlation between disease severity and fc levels and found a non-significant higher mean stool fc level in patients with severe disease, although in this study a different severity scoring system was used [16].

The main limitation of our study was the small sample size, reflecting the fact that our medical center is a 300 bed hospital serving only the North-East of Israel and thus we do not have many patients with community acquired CDI, and due to rigorous infection control programs we do not have many cases of nosocomial CDI as well. The patients in this paper represent all patients with CDI at our hospital during one year. A larger sample size might have helped us find a stronger correlation between fc levels and disease severity and outcomes.

## Conclusions

Finding novel and easy performance tools for the assessment of CDI severity is an important and crucial goal. Our study highlights the fact that fc has a potential role as a biomarker of the disease severity and binary toxin producing ribotype-associated disease.

## Methods

### Patients

Overall, 29 patients with diarrhea, who were found positive for *C. difficile* from June 2014 to May 2015, were admitted at the Poria Medical Center located in northern Israel. Identification of *C. difficile* from patients’ stool specimens was carried out on a bio-molecular platform, which also enables identification of *C. difficile* ribotype 027 (Xpert® *C. difficile,* Cepheid, Sweden) [19]. The following demographic and clinical data concerning patients with CDI were collected from electronic files: age, sex, functional status, community versus nosocomial acquired CDI, and antibiotic susceptibility.

A clostridium severity score index was calculated. For each patient the score incorporated nine parameters, each variable added one point: altered mental status, abdominal pain or distention, 1500 > WBC > 20,000, ALB < 2.5, ascites or colitis (imaging), MAP < 65, pulse > 110, ICU transfer. A score of 0–3 criteria meant mild disease, 4–6: moderate disease, ≥7: severe disease [10].

The study protocol was reviewed and approved by the Poria-Baruch Padeh Medical Centre Institutional Review Board/Ethics Committee. A waiver was ushered for informed patient consent.

### Fecal calprotectin test

Calprotectin levels in stool were examined by Liaison® Calprotectin Stool (Saluggia, Italy) kit using Chemiluminescent immunoassay (CLIA).

### Bacterial culture and determination of in vitro susceptibility

Antibiotic susceptibility test was performed by culture method of the stool sample on chromID™ *C. difficile* (bioMérieux, Durham, NC) growth medium and then incubated at 37 °C in anaerobic conditions for 18–12 h. *C. difficile* colonies appear as asymmetric and black-colored colonies [20]. One isolated *C. difficile* colony from the culture was suspended in Thioglycollate broth (Becton Dickinson, Heidelberg, Germany (until density of 1.0 McFarland was reached. The inoculum was spread by cotton swab on Brucella Blood Agar growth medium, supplemented with haemin and vitamin K (Becton Dickinson, Heidelberg, Germany (. MIC determination for metronidazole and vancomycin was performed by a gradient Etest strip (bioMérieux, Durham, NC). Further, antibiotic susceptibility plates were incubated in anaerobic conditions at 37 °C for 24 h. The interpretation of susceptibility test results was performed in accordance with European Committee on Antimicrobial Susceptibility Testing (EUCAST) recommendations. Based on these recommendations, *C. difficile* isolates are considered as strains with reduced susceptibility values to metronidazole or vancomycin when MIC level >2 mg/L [21].

### Statistical analysis

Chi-square test was applied for analyzing the differences in categorical parameters by NAP-1. The Non-parametric Wilcoxon-Mann–Whitney Rank sum was applied for analyzing the difference in the quantitative parameters by NAP-1, and for analyzing differences in calprotectin levels between categorical variables (recurrence, reduced antibiotic susceptibility, death, gender, nursing house, function, and confusion). Pearson correlation was used for testing the association between calprotectin and quantitative parameters for testing the association between calprotectin and categorical parameters. Analysis of Covariance (ANCOVA) model was applied for analyzing the different calprotectin levels by NAP-1 with adjustment to confusion, index-1, and age. All tests applied were two-tailed, and a *p* value of 5 % or less was considered statistically significant. The data was analyzed using SAS® version 9.3 (SAS Institute, Cary, North Carolina).

## Abbreviations

CDI, *Clostridium difficile* infection; Fc, fecal calprotectin
